# Avoid jumping to conclusions under uncertainty in Obsessive Compulsive Disorder

**DOI:** 10.1371/journal.pone.0225970

**Published:** 2020-01-15

**Authors:** Sharon Morein-Zamir, Sonia Shapher, Julia Gasull-Camos, Naomi A. Fineberg, Trevor W. Robbins

**Affiliations:** 1 School of Psychology and Sports Science, Anglia Ruskin University, Cambridge, United Kingdom; 2 Behavioural and Clinical Neuroscience Institute; Department of Psychology, Cambridge University, Cambridge, United Kingdom; 3 Hertfordshire Partnership University NHS Foundation Trust, NHS; University of Hertfordshire, Hatfield, United Kingdom; University of Wuerzburg, GERMANY

## Abstract

High levels of intolerance of uncertainty (IU) could contribute to abnormal decision making in uncertain situations. Patients with Obsessive Compulsive Disorder (OCD) often report high IU, indecisiveness and the need to seek greater certainty before making decisions. The Beads task is a commonly used task assessing the degree of information gathering prior to making a decision and so would be predicted to show impairments in OCD patients. Results to date have found mixed support for this, possibility due to methodological issues. Here, a group of OCD patients (n = 50) with no comorbidities was compared with age, gender, and verbal-IQ matched controls (n = 50) on the most commonly used version of the Beads task. An independent sample of healthy volunteers with high versus low OC symptoms, and high versus low IU were also assessed (n = 125). There was no evidence that patients with OCD differed from control volunteers in the degree of information gathering prior to making a decision. Medication status and age did not appear to mediate performance. Similarly, there were no association in healthy volunteers between task performance and OC or IU characteristics. Additional measures examining the degree of certainty initially showed support for greater uncertainty in patients, but this was due to deviations from task instructions in a subset of patients. We conclude that despite the large sample size and good matching between groups, the Beads task in its most widely used form is not a useful measure of IU or of information gathering in OCD. The results argue against a robust behavioural difference in OCD when compared to controls. Recommendations for future studies employing the task are discussed.

## Introduction

Obsessive-compulsive disorder (OCD), with a lifetime prevalence of 2–3%, is characterized by recurrent intrusive thoughts, and ritualistic repetitive behaviours or mental acts [1]. The disorder is associated with substantial personal distress and societal costs [2]. Clinical impression of everyday impairments in patients include severe difficulties making decisions, most typically characterized by indecisiveness and pathological doubt [3]. This is seen even in contexts unrelated to their obsessions and compulsions [4]. However, research using formalized cognitive testing has not shown consistent evidence for decision making difficulties in OCD patients [5, 6]. One aspect of decision making that could lead to everyday impairments is high levels of intolerance of uncertainty, whereby individuals perceive and respond abnormally in uncertain situations [7].

High intolerance of uncertainty (IU) refers to experiencing doubt as aversive, with even moderate uncertainty being experienced as stressful and upsetting [7, 8]. Initially developed in relation to anxiety, it is increasingly posited as a transdiagnostic construct [9]. Notably, patients with OCD report elevated IU, with a positive association between IU and OC traits and behaviours [10, 11]. It may be this characteristic in OCD that is linked to indecisiveness, being overly cautious and requiring excessive deliberation, as individuals strive to accumulate more evidence before making decisions.

In accordance with this notion, patient performance has been investigated in tasks assessing information gathering prior to making a decision (e.g., [12–14]). The most prominent has been a probabilistic inference/reasoning task, known as the Beads task [15]. Participants view two containers, each holding a mixture of two bead colours (or balls/straws). Typically each container, or jar, contains mostly beads of one colour. Participants are asked to decide which of the two jars has been selected (by the experimenter or computer). To do this, they can request beads one at a time from the selected jar, until they are ready to make their choice. In this version (Condition 1), the key outcome measure is draws to decision, which is hypothesized to be greater in OCD patients. Other versions require probabilistic estimates from the participant on each draw, this being sensitive to how participants shift their judgements of certainty. Namely, a fixed number of beads is drawn from the selected jar and after each draw, participants estimate the relative likelihood of each jar having been selected (Condition 2). Here it is hypothesized that OCD patients will exhibit less extreme probability estimates, reflecting reduced certainty. They may also exhibit greater departures from an objective observer often calculated as Bayesian normative values when compared to controls [16, 17]. These performance indices should provide objective behavioural measures of IU in OCD in a controlled setting.

Initial evidence supported the hypothesis that OCD patients indeed require more beads before making a decision ([15, 16, 18] see also [19]). Several subsequent studies however, failed to support this [20, 21]. More recently, one study provided evidence for the opposite notion, with OCD patients requesting *fewer* beads than controls [22]. This was interpreted as greater impulsivity in these patients, as also evidenced by higher self-reported impulsivity (see also [23]). These patients presumably adopted an impulsive response style, requiring less information and even jumping to conclusions [22]. Jumping to conclusions (JTC) reasoning style, whereby participants ask for only one or two beads before making their decisions, has been reliably captured by the Beads task in patients with delusions and is believed to contribute to the formation and maintenance their symptoms [24, 25]. Mixed results pertaining to OCD performance in evidence seeking tasks is not specific to the Beads task, being reported in the Information Sampling Task where excessive draws to decision were found in some studies but not others [6, 13]. These latter studies raise the possibility that age may mediate draws to decision, as positive results were found in young but not older samples [26].

Closer inspection of studies employing the Beads task with OCD samples reveals a complex picture (see also Table 1). Studies vary in sample characteristics, and sample size has often been modest, with three of seven studies reporting on 12 or fewer patients. The presence of comorbid disorders may contribute to the inconsistent findings, as a greater number of draws has been associated with disorders such as bulimia nervosa [27]. Conversely, binge drinking, schizophrenia and delusional disorders are associated with fewer draws [28, 29]. Medication may also play a role, as indirect catecholaminergic agonist drugs, such as methylphenidate, can alter task performance, although in ways dependent on baseline performance [30]. Moreover, reduced draws were reported in a sample of mostly treatment refractory OCD patients receiving both serotonin reuptake inhibitors (SSRI) and antipsychotic medication, who were previously unresponsive to SSRI treatment thus suggesting drug-related effects [22]. Conversely, positive findings were found in OCD patients who had undergone deep brain stimulation, though this sample is not representative consisting of severe and largely treatment-resistant patients [19].

**Table 1:** Summary of previous studies of beads task in OCD.

Study	Participants	Comorbidities	Medication Status	Condition 1 findings^a^	Condition 2 findings^b^	Note
Volans (1976)	8 Obsessionals; 8 HC	n/a	n/a	+ after neuroticism partialed out	Greater deviation from Bayesian norm on first draw	22 phobic patients
Fear & Healy (1997)	26 OCD; 30 HC	n/a	n/a	n.s. Draws 3.4 in OCD and 2.6 in HC	OCD under-confident, less certain and deliberated longer	22 DD; 15 OCD+DD
Pélissier & O’Connor (2002)	12 OCD; 12 HC	No comorbidities	n/a	(+) Draws 11.0 in OCD and 7.56 in HC. More decision errors (+)	n.s.	10 GAD
Reese et al. (2011)	20 OCD; 20 HC	10 with comorbidities	15 medicated	n.s. OCD adjust less to difficulty changes	n/a	20 BDD
Jacobsen et al. (2012)	32 OCD; 16 HC	No comorbid psychotic disorders, other comorbidities n/a	n/a	n.s. Draws 4.82 in OCD and 3.88 in HC	n/a	OCD patients with high versus low conviction; 16 with delusions
Grassi et al (2015)	38 OCD; 39 HC	No comorbidities	Majority (32) medicated	- Draws 3.76 in OCD and 7.79 in HC	n/a	27 treatment resistant
Voon et al (2017)	12 OCD; 24 HC	2 with comorbidities	All medicated	+ Draws 12.55 in OCD and 5.49 in HC	n/a	All treatment resistant & undergone DBS

OCD, Obsessive-Compulsive Disorder; HC, healthy controls; DD, delusional disorder; DBS, deep brain stimulation; GAD, generalized anxiety disorder; BDD, body dysmorphic disorder.

^a^Condition 1: procedures where participants respond by merely asking for additional beads or not.

^b^Condition 2: procedures where probabilistic estimations or decisions are required.

Key experimental and procedural characteristics likely also contribute to the diversity of findings. For example, in some studies participants requested beads directly from the experimenter, raising the possibility of experimenter bias [15, 16, 18, 20]. The limited availability of information from previous draws may also have biased some results, as this can increase working memory demands and contribute to indecision. The use of different beads sequences, specifying the positions of non-majority colour beads, could also influence task sensitivity. While some have used established sequences (e.g., [17, 31]), others have used alternative sequences or have not specified the sequence used.

The sequence is particularly important in Condition 2. The commonly used fixed sequence of 20 beads involves a reversal of the dominant colour mid-way, allowing researchers to investigate biases in schizophrenic patients [17]. OCD patients may have abnormalities in reversal learning [32] and tend to be overly rigid in their response patterns [33]. It has also been suggested that Condition 2 is more sensitive because it is more cognitively demanding and consequently more stressful, making it more likely to capture abnormal decisional processes [15, 16]. However, here too findings are inconsistent [16, 18]. Additionally, participants in Condition 2 may not always fully understand the instructions, making their estimation on the most recent bead or forgetting to take into account that the entire sequence is drawn from one jar [34, 35]. This miscomprehension may underlie a pattern of responding labelled over-adjusting, believed to capture radical changes in decisions in the face of modest accumulations of disconfirming evidence [17, 34].

These procedural considerations are also of relevance when assessing the hypothesized link between Beads task performance and IU, regardless of OCD. Preliminary evidence indicated a positive association between beads requested and self-reported IU in students [36]. A subsequent study of anxiety patients (a third of which met diagnostic criteria for OCD) and controls did not detect behavioural differences [37]. Similarly, no associations between IU and Beads task performance were found in community or eating disorder samples [3, 27]. An alternate hypothesis put forward is that high trait anxiety individuals may gather less evidence before deciding, in an effort to shorten the duration of experiencing uncertainty [38]. Though IU was not directly assessed, those with high- compared to low-trait anxiety requested fewer beads, seemingly indicating a JTC reasoning style, and chose the incorrect jar more often, suggesting an implicit motivation to shorten the uncertain state even at the expense of correctness [38]. This scenario could account in part for the JTC style found in a sample of largely treatment refractory OCD patients [22].

The present study set out to replicate one of the most commonly used procedures of the Beads task, assessing performance in both conditions 1 and 2, in a fully computerized task where the experimenter did not mediate responding. The patients were largely without comorbidities, thus ensuring we could assess the role of OCD specifically. Similarly, the large sample size enabled us to assess the possible role of medication status. Given that OC traits occur in the general population and are dimensional in nature [39], an additional non-clinical student sample was investigated. OC symptoms, IU and anxiety were collected to assess their relative associations with performance.

## Methods and materials

### Study 1: OCD patient and control sample

Control participants had no current or past psychiatric disorders as determined by a screening interview including the MINI [40], and were not taking any psychoactive medications. Patients met DSM-IV criteria for OCD with no other axis-I disorders as determined by a detailed structured clinical interview with a psychiatrist. Following testing it was ascertained that three patients had met criteria for Generalized Anxiety Disorder and one for Anorexia Nervosa. Inclusion of these patients did not alter the results so they were retained. Exclusion criteria for all participants included current or past neurological disorders or brain damage. Of the 50 patients, 18 were unmedicated, 29 were prescribed SSRIs with six prescribed an adjunct antipsychotic. Two also received an adjunct antidepressant, one patient was prescribed pregabalin and one lithium carbonate. The study received NRES Cambridgeshire Research Ethics Committee approval (10/H0308/27), and participants provided written informed consent, receiving a small financial reimbursement for taking part.

### Study 2: Student sample

Participants were recruited from adverts and an undergraduate participant pool. Exclusion criteria included current or past diagnosed psychiatric disorder, neurological disorders or brain damage. The study was approved by the Cambridge University Psychology Ethics Committee. Participants provided written informed consent and received either course credit or a small financial reimbursement.

### Procedure and materials

Participants in both samples were seated at a comfortable viewing distance and tested individually with the experimenter present. For patients, prior to testing, symptom severity was assessed with the Yale-Brown Obsessive-Compulsive Scale (YBOCS, [41]) and depression symptom severity was assessed with the Montgomery-Asberg Depression Rating Scale (MADRS, [42]). Participants completed the following questionnaires with Latin square counterbalancing: Obsessive Compulsive Inventory (OCI, [43]), state/trait Anxiety Inventory (STAI, [44]), Barrett Impulsivity Scale (BIS, [45]) and Intolerance of Uncertainty (IU, [46]). IU included all 27 original items, but following recent evidence supporting the 12-item two-factor model [47, 48], scores were computed for Prospective and Inhibitory IU [49]. Twenty student participants did not complete the IU and BIS due to experimenter error. Verbal IQ was assessed with the National Adult Reading Test (NART, [50]). The Beck Depression Inventory, Padua Inventory and Metacognitive Questionnaire were administered to some participants but not analyzed [51–53]. Participants performed additional tasks not reported here.

**Fig 1:**
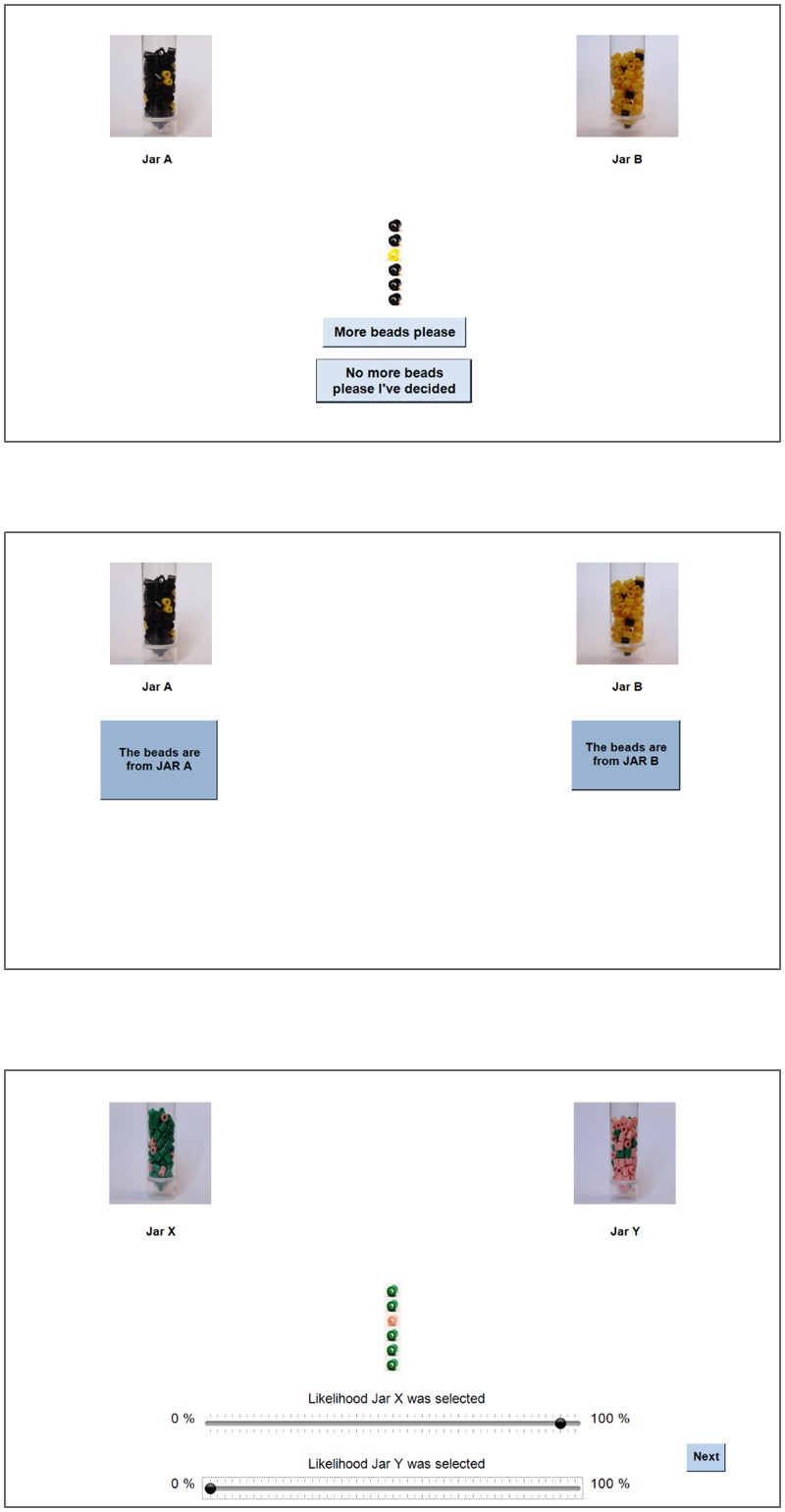
Beads task. Participants view the two jars in Condition 1, with options for requesting more beads or not. The results of all draws to date are presented in the middle, with a bead being added after each request (panel A). Participants’ view in Condition 1 before making their final decision between Jar A and Jar B (panel B). Example of Condition 2 following the 6^th^ draw, after making likelihood estimations that the beads were drawn from Jar X or Jar Y (panel C).

The Beads task consisted of two conditions and was based on previous publications with the same order of draws for Condition 1: AAABAAAAABBAAAAAAAAB, and Condition 2: AAABAAAABABBBABBBBBAB [16, 17]. Condition 1 showed two jars with a mixture of yellow and black beads, one with 85% yellow and the other with 85% black beads. Images of the jars were present in the instructions and throughout the task. The selected jar, determining bead sequence and its location, was counterbalanced within each group. Instructions first informed participants that they had to decide from which of the two jars the beads are drawn. They explicitly stated that beads will be taken from the same jar for all draws; the beads will be replaced back to the same jar after each draw and that participants could request as many draws as they wished so as to be completely sure which jar was chosen. They were then questioned as to the chance that the first draw, from either jar would be black. If unclear, the experimenter explained the instructions and clarified the question. Subsequently, the experimenter remained present, out of line of sight throughout, and did not interfere other than to answer questions and clarify instructions. Participants then could draw beads from the chosen jar one at a time. After each draw two options appeared, and participants chose between ‘More beads please’ and ‘No more beads please I’ve decided’ (Fig 1A). If more beads were requested, a bead was added to the sequence. All draws remained on screen in the order drawn, removing any reliance on working memory. Alternatively, buttons appeared below each jar and the participant could make their decision (Fig 1B).

Condition 2 (probabilistic estimation) presented green and pink beads, with the jars labelled X and Y, and always followed Condition 1. Instructions were similar but stated that participants will now see beads being drawn for them from either jar X or Y and that they had to decide from which jar the beads were drawn. For ease of communication to the reader jar A and X will refer to the jar corresponding to the majority of beads in the first 10 draws though in practice jar chosen and its location were counterbalanced. On each of 20 draws, the sequence to that point appeared on the screen. After 1.5 sec, two horizontal scales appeared (labelled X and Y, respectively) and participants had to estimate the likelihood of each jar having been selected (Fig 1C). The scales were adjusted independently and coded on a 50 point scale [35]. The ‘next’ button allowing participants to proceed to the next draw appeared only after adjusting both scales, which were initially midway. Two scales, rather than one, allow independence of probability estimates and no loss of information due to imposed reciprocity [35]. In both conditions no time limits were placed on responses, the sequence of drawn beads remained on screen throughout, and no feedback was provided. The experiment, programmed using Visual Studio, was conducted on a 15.6” laptop with external mouse, running Windows 7.

### Analyses and design

Analyses probed group differences between patients and controls. For comparability and to facilitate descriptive statistics, similar analyses were conducted on the student sample using a median split on total OCI score, along with correlational analyses. Given the hypothesized importance of IU, secondary analyses employed a median split on IU. In Condition 1 of the task, the mean number of draws to decision and jar chosen were assessed, as was mean deliberation time and JTC style, defined as requesting one or two beads. In Condition 2 of the task, probabilities estimated for each draw were assessed as in previous studies [17, 18]. These include the following, with the number in parentheses referring to the index as provided by Fear and Healy (1997): a) *Initial posterior estimate (4)*: The estimate of the likelihood of Jar X being chosen after draw 1. The Bayesian estimate is 85%, scores below represent under-confidence and above represent over-confidence. b) *Draws to certainty (5)*: The number of draws to an estimate of 100%, or if not reached, two estimates of 85%. c) *Effect of confirmatory evidence on subsequent judgement (6a)*: The increase in the second from the first posterior estimate, with a positive score showing an increase in confidence following confirmatory evidence. d) *Effect of disconfirmatory evidence on subsequent judgement (6b*): The decrease in the fourth from the third posterior estimate, calculated as posterior estimate from draw 3 minus draw 4 so that a positive score indicates decreased confidence. e) *Errors in decision making*, *after draw 10 (7)*: A correct decision is taken to be a probability greater than 85% favoring jar X. f) *Draws from draw 10 to change in estimate (8)*: Number of draws from draw 10 to any change, indicating the number of items to a change having reached a decision. g) *Size of first estimate change (9)*: Absolute difference in size of estimate at draw 10 and first point of change. h) *Final decision (10)*: Estimate after draw 20 of jar X being chosen. i) *Mean time taken per draw estimations (11)*. To assess behaviour against that predicted by a Bayesian ideal observer, deviations between participant estimates and the Bayesian norm were calculated for Condition 2 and subjected to an ANOVA with the factors of group, jar and draw.

Initial inspection of the data supported the notion that some participants did not fully apprehend the instructions in Condition 2 as has been previously reported [34]. Miscomprehension was defined as estimating the beads coming from jar Y where the sequence clearly indicated jar X, which in practice entailed a lower rating for jar X than jar Y in draws 4 or 9. This was in keeping with the findings regarding ‘extreme over adjustment’ [34]. Excluding these participants resulted in analyses of Condition 2 being conducted on 35 patients, 40 HC and 53 and 55 participants in the low- and high-OCI groups, respectively. Group characteristics of the smaller samples remained similar to the original samples (see also Table A in S1 File).

Group comparisons were assessed using between subjects t-tests and non-parametric Mann-Whitney when outliers or departures from normality were detected. Deviations from the Bayesian norm were assessed with a mixed ANOVA. Secondary analyses assessed the role of medication in patients. Group differences are reported without control for type I error to strengthen the conclusions regarding no significant effects, with r and Cohen’s d effect sizes reported where relevant. Pearson correlations probed associations between task performance and self-report indices and were Bonferroni corrected for type I error.

## Results

### Demographic and clinical characteristics

OCD patients and control participants in Study 1 did not significantly differ in age, verbal intelligence or gender distribution (Table 2). OCD patients had moderate symptom severity and relatively low depression scores. Patients did not differ in motor and non-planning impulsivity but reported worse attention impulsivity, anxiety, and IU.

**Table 2:** Means and standard deviations of control and OCD patient group characteristics.

		Controls (n = 50)	OCD (n = 50)		
Characteristic	Measure	M (SD)	M (SD)	*Z*	*p*
Gender	M:F	26:24	27:23		
Age	Years	38.44 (14.29)	41.46 (14.31)	1.03	0.30
Verbal IQ	NART	115.87 (7.43)	115.29 (5.98)	0.40	0.68
Obsessions & Compulsion	YBOCS		20.49 (5.34)		
Depression	MADRS		7.33 (5.91)		
Impulsivity- attention	BIS- attention	14.44 (3.26)	17.94 (4.45)	4.37	<0.001
Impulsivity–motor	BIS—motor	21.74 (3.81)	20.55 (5.12)	1.40	0.16
Impulsivity–non planning	BIS–non planning	23.88 (4.92)	23.31 (6.09)	0.78	0.43
State Anxiety	STAI-S	31.22 (9.69)	43.70 (12.13)	5.11	<0.001
Trait Anxiety	STAI-T	35.82 (10.07)	57.70 (11.57)	7.14	<0.001
IU27	IU	54.32 (15.64)	85.00 (23.28)	6.22	<0.001
Prospective IU	IU	17.14 (4.58)	24.23 (6.80)	5.24	<0.001
Inhibitory IU	IU	7.96 (3.24)	14.49 (4.72)	6.32	<0.001
Obsessions & Compulsion	OCI	9.80 (7.58)	33.14 (11.28)	7.77	<0.001

Note. NART: National Adult Reading Test; YBOCS: Yale-Brown Obsessive Compulsive Scale; MADRS: Montgomery-Asberg Depression Rating Scale; BIS: Barret Impulsivity Scale; STAI-S: State/Trait Anxiety Inventory-State; STAI-T: State/Trait Anxiety Inventory-Trait; IU: Intolerance of Uncertainty; OCI: Obsessive Compulsive Inventory-Revised;

NART scores available for 41 controls and 42 patients. MADRS scores available for 42 patients;

Table 3 describes the demographic and self-report characteristics of low- and high-OCI groups in the student cohort in study 2 as determined by a median split on OCI scores (*Mdn* = 12.5). The groups did not differ in age, gender or verbal intelligence, with high-OCI participants scoring higher on self-reported anxiety, IU and most impulsivity measures.

**Table 3:** Means and standard deviations of Low and High OCI participants.

		Low OCI (n = 62)	High OCI (n = 63)		
Characteristic	Measure	M (SD)	M (SD)	Z	*p*
Gender	M:F	17:43	18:45		
Age	Years	24.08 (4.31)	23.21 (3.23)	0.9	0.35
Verbal IQ	NART	114.99 (8.94)	112.47 (8.92)	1.45	0.15
Impulsivity- attention	BIS- attention	15.26 (3.29)	17.81 (3.54)	3.13	0.002
Impulsivity–motor	BIS—motor	21.87 (3.19)	23.41 (4.09)	1.65	0.10
Impulsivity–non planning	BIS–non planning	21.56 (4.35)	23.84 (5.26)	2.06	0.04
State Anxiety	STAI-S	30.66 (7.33)	39.08 (11.68)	4.26	<0.001
Trait Anxiety	STAI-T	35.50 (7.63)	46.96 (12.21)	5.50	<0.001
IU27	IU	52.46 (13.72)	77.57 (20.52)	6.08	<0.001
Prospective IU	IU	15.79 (4.93)	21.50 (6.09)	4.44	<0.001
Inhibitory IU	IU	7.58 (3.02)	12.94 (4.50)	6.25	<0.001
Obsessions & Compulsion	OCI	6.69 (3.73)	24.60 (11.23)	9.64	<0.001

Note. NART: National Adult Reading Test; YBOCS: Yale-Brown Obsessive Compulsive Scale; MADRS: Montgomery-Asberg Depression Rating Scale; BIS: Barret Impulsivity Scale; STAI-S: State/Trait Anxiety Inventory-State; STAI-T: State/Trait Anxiety Inventory-Trait; IU: Intolerance of Uncertainty; OCI: Obsessive Compulsive Inventory-Revised.

BIS scores available for 39 and 32 Low and High OCI participants, respectively. IU scores available for 48 and56 Low and High OCI participants, respectively.

### Task Condition 1

Patients (5.24, SD = 3.29) did not differ from controls (5.96, SD = 3.90) in draws to decision (*Z* = 1.00, *U* = 1104.5, *p* = 0.32, d = 0.20). There was also no significant difference in the proportion in each group who chose after only one or two draws (χ^2^(1) = 1.10, *p* = 0.29; 6 patients vs. 3 controls). Nor did patients and controls differ in choosing the jar corresponding to the first bead (χ^2^(1) = 0.34, p = 0.56; 96% vs. 98%, respectively). Additionally, mean deliberation time per draw did not differ between patients and controls (*t*(98) = 0.86, *p* = 0.39, d = 0.17; 9.95 sec vs 8.73 sec, respectively). In the student cohort, high-OCI (5.62, SD = 3.34) did not differ from low-OCI (5.03, SD = 2.04) in draws to decision (*Z* = 0.58, *U* = 1836.5, *p* = 0.57, d = 0.21). JTC bias was rare, with two and one participants demonstrating this reasoning style in the high- and low-OCI groups respectively (χ2(1) = 0.33, p = 0.57). Almost everyone in both groups chose the jar corresponding to the first bead (χ^2^(1)<0.01, *p* = .99) with two choosing incorrectly in each group. Finally, mean deliberation time per draw did not differ between high- and low-OCI participants (*t*(123) = 0.35, *p* = 0.73, d = 0.06; 6.87 sec vs 6.63 sec, respectively).

To assess support for the hypothesis that there were no group differences, Bayes factors were computed in JASP (JASP Team, 2018). Using a two-sided Bayesian hypothesis test (default Cauchy prior width of r = 0.707), Condition 1 results indicated moderate support for the null hypothesis when taken separately, BF_01_ = 2.78 and BF_01_ = 3.05 for Studies 1 and 2 respectively. The posterior median was 0.18 and a 95% credible interval from -0.19 to 0.55 for Study 1 and posterior median of -0.19 and a 95% credible interval of -0.53 to 0.16 for Study 2. The use of the “Oosterwijk prior” also did not provide support for the alternative hypothesis in either study.

### Task Condition 2

Group comparisons indicated that patients and controls were similar in performance with no significant differences on any outcome measure (see Table 4). In the student sample, contrasting low- and high-OCI participants showed similar results with no significant differences. An exception to this was the size of the first estimate change after draw 10, though given the overall findings and lack of type I error correction, this was taken to be a type I error. Results from the full cohorts are presented in the SI (see Table B in S1 File).

**Table 4:** Dependent variables and *p* values for task Condition 2.

	Study 1			Study 2		
	Controls (n = 40)	OCD (n = 35)	*p*	Low OCI (n = 53)	High OCI (n = 55)	*p*
Initial posterior estimate (4)	69.35 (17.01)	73.31 (16.27)	.31	76.94 (12.35)	73.64 (16.39)	.24
Number of draws to certainty (5)**	6.43 (4.28)	5.50 (2.33)	.29	4.96 (2.25)	5.64 (3.54)	.25
Confirmatory effect (6a)	8.05 (11.58)	5.20 (15.84)	.37	8.83 (7.94)	6.58 (11.34)	.24
Disconfirmatory effect (6b)	1.60 (10.72)	-0.29 (15.16)	.53	3.17 (4.63)	1.60 (15.69)	.49
Percent errors at draw ten (7)*	10.00%	17.14%	.36	3.77%	10.91%	.16
Number of draws from ten to change (8)	2.59 (2.38)	1.88 (1.65)	.11	2.22 (1.54)	2.56 (1.87)	.16
Size of first estimate change (9)	12.16 (19.49)	7.94 (10.72)	.28	5.16 (7.76)	11.42 (19.36)	<.01
Percent final decision Jar X (10)*	77.50%	82.86%	.57	83.02%	81.81%	.87
Mean time in seconds per draw decision (11)	12.45 (3.76)	14.27 (8.17)	.21	11.57 (5.30)	10.84 (3.05)	.38

Note.

*Percentage of participants, p denoting Chi-squared test;

**Sample size for this measure were 37 and 30 for controls and patients, respectively; 51 and 50 for low- and high-OCI participants, respectively.

**Fig 2:**
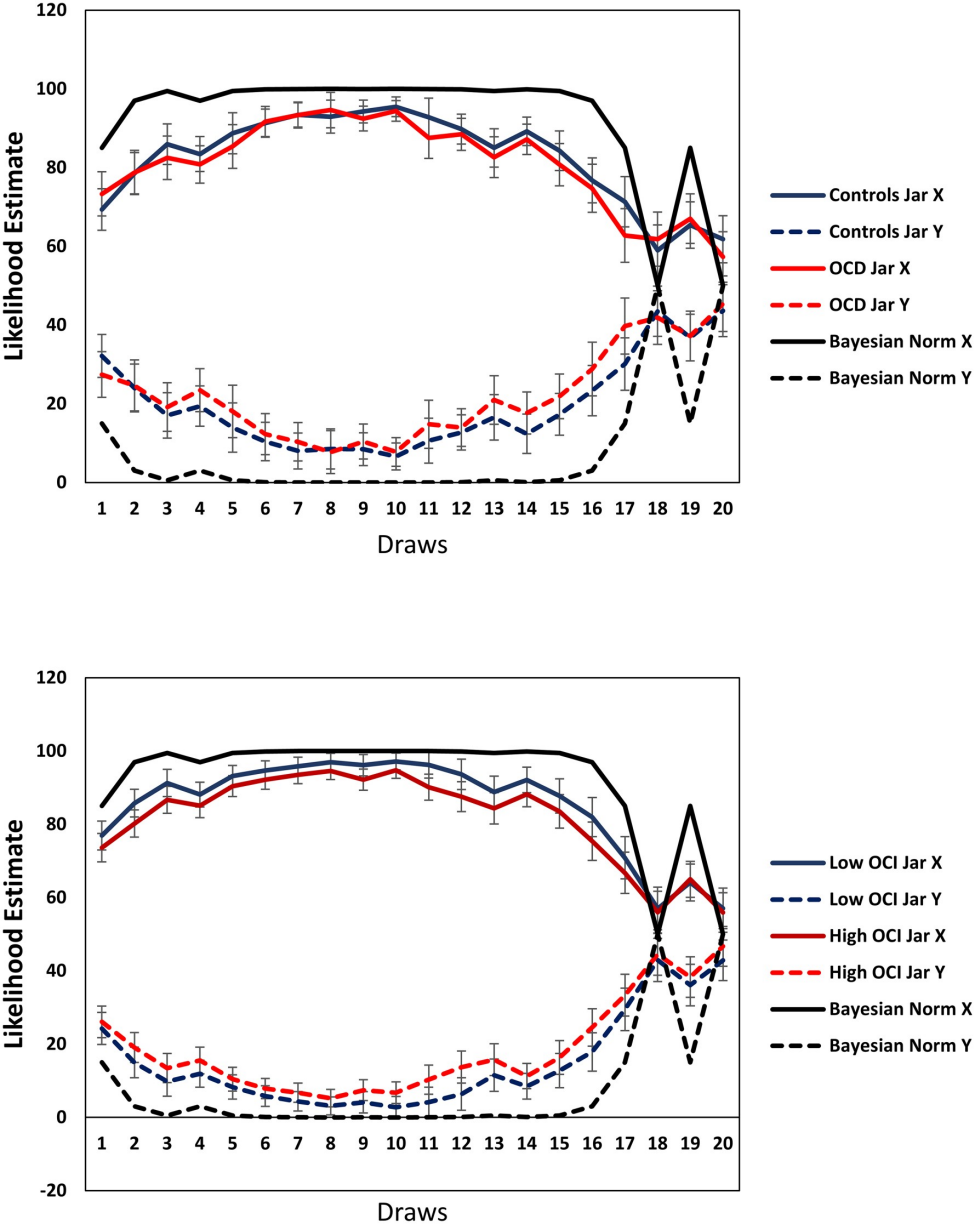
Mean probabilities estimated for each draw of Condition 2 by group for each jar, together with the Bayesian normative values. Fig 2a depicts values for OCD and control groups. Fig 2b depicts values for low-OCI and high-OCI groups.

Fig 2 shows the mean probabilities estimated for each draw of Condition 2 by group for each jar, together with Bayesian normative values. The latter demonstrates a rise to certainty for jar X within two draws, remaining high until draw 16 before settling on 50% for both jars, in accordance with the final tally of beads. Both patients and controls follow this pattern, but show decreased certainty for both jars compared to the Bayesian normative values. Importantly, there is little evidence for group differences (Fig 2a). Similarly, there was no evidence for differences between low- and high-OCI participants in their likelihood estimations (Fig 2b).

An ANOVA on patients and controls in Study 1 assessing mean deviation from the Bayesian norm for each draw by group and jar indicated no effect for group (*F*(1,73) = 0.73, *p* = .39), nor did group enter into any interactions (*p*’s>.32). There was a main effect for jar such that the deviations were greater for jar Y (13.14) compared to jar X overall (10.51), (*F*(1,73) = 9.26, *p* = .003). Additionally, deviations from the norm varied with draw number (*F*(19,1387) = 32.99, *p*<.001), largely demonstrating under-confidence throughout, which gradually reduced towards draw 10 before rising again, but also over-confidence in draws 18 and 20. The equivalent ANOVA on the student sample in Study 2 revealed marginal effects for group (*F*(1,106) = 3.66, *p* = .058), such that deviations from the Bayesian norm were larger for the high-OCI (10.60) compared to the low-OCI group (7.08). There was also a marginal effect for jar (*F*(1,106) = 2.99, *p* = .086) with greater deviations for jar Y (9.06) compared to jar X overall (8.62). Deviations over draws followed a pattern similar to that seen in patients and controls (*F*(19,2014) = 36.86, *p*<.001). No other effect was significant (*p*’s>.41).

Of note, analyses including all participants from Study 1, regardless of evidence supporting misapprehension of the instructions, indicated that the OCD patient group showed a greater departure compared to controls from Bayesian normative values for both jars. This was particularly apparent for the draws involving beads from jar Y and from draw 10 onwards (see SI). This pattern was not evidenced when analyzing all participants in Study 2.

### Correlation analysis

Correlations explored possible associations between task performance and demographic, clinical or questionnaire variables. Associations were visually inspected for non-linear relationships and outliers. Number of draws did not correlate with any variables in either sample, nor within the patient group (all r values<|.1|). Mean deliberation time demonstrated weak positive associations in Study 1 (both patients and HC) with age (*r*(98) = 0.21, *p* = .033), IU (prospective IU: *r*(98) = 0.26, *p* = .010; inhibitory IU, *r*(98) = 0.21, *p* = .042) and trait anxiety (*r*(98) = 0.20, *p* = .044). These associations were also present in the patients alone (*r*(48) = 0.31, *p* = .030, *r*(48) = 0.46, *p* = .001 and *r*(48) = 0.32, *p* = .028, *r*(48) = 0.40, *p* = .004, for age, prospective IU, inhibitory IU, and trait anxiety, respectively). In the patient sample, similar positive associations were noted for YBOCS (*r*(48) = 0.39, *p* = .006), and OCI (*r*(48) = 0.33, *p* = .021). With Bonferroni corrections, only associations between mean deliberation time and prospective IU, trait anxiety and YBOCS remained significant. In the student cohort there was only a weak association with age (*r*(122) = 0.18, *p* = .042).

### Secondary analyses

We explored the role of medication by contrasting medicated (n = 32) and unmedicated patients (n = 18) with their respective control groups, which were matched for age, gender and verbal IQ. The analyses for Condition 2 (excluding participants as detailed above) retained 20 and 15 patients in the medicated and unmedicated groups, respectively. No significant differences in performance were noted for any comparison in either Condition 1 or 2. The two patient groups differed in age (*t*(48) = 2.75, *p* = .008), with medicated (45.38, SD = 13.72) being older than unmedicated patients (34.50, SD = 12.91). Additionally, medicated patients scored higher on the BIS inattention subscale (*t*(48) = 2.72, *p* = .009). Given the hypothesized role of IU, we analysed student task performance in Study 2, this time using a median split on total IU rather than OC symptoms. This did not reveal significant differences, replicating the results from the analyses for both conditions reported above.

## Discussion

Given the potential utility for an objective performance index for IU in OCD, this study set out to replicate one of the most commonly used procedures of the Beads task, assessing performance in both Conditions 1 and 2. We took care to address concerns regarding potential task-related confounds, including experimenter bias, working memory load, and miscomprehension. Patients were largely without comorbidities ensuring we could assess the specific role of OCD, and sample size allowed for secondary evaluation of the role of medication status. The results indicated no evidence for OC symptoms or IU playing a role in performance in one of the most commonly employed versions of the task. Despite clear group differences in self-reported traits, findings did not support the notion that patients required increased evidence before making their decision. A second sample of 125 individuals, capitalizing on the dimensional nature of these traits [39], revealed similar findings, with no association between OC or IU traits and beads performance. Together, the results support the conclusion that the Beads task, at least in its present configuration is insensitive to both indecisiveness as manifested in OCD, and to IU.

There was a weak positive association between mean deliberation time and IU and anxiety. This was evident particularly in patients, where those with worse OC symptoms required longer to decide whether to request more information. This tendency is consistent with the clinical impression of patients being cautious and hesitant, requiring lengthy amounts of time to make decisions in everyday life [4, 16]. Notably though, this was not found in the group comparison or in the healthy volunteer cohort. Deliberation time in decision making tasks is not routinely reported in OCD studies, although some have reported patients being slower under specific conditions [4, 12, 54]. Moreover, greater deliberation times can be found in OCD on a host of cognitive tasks, limiting any interpretation of such findings [55]. Importantly, although longer decision time is characteristic of indecisiveness, indecision goes beyond not making timely decisions [56]. Future studies may explore conditions when deliberation times under uncertainty might be linked to more direct behavioural measures of seeking certainty.

The results of the present samples show that patients, those characterized by high OC traits or high IU can make decisions under some circumstances without resorting to greater information gathering. Taken together with previous findings, it can be concluded that the Beads task, in its present format, is not a sensitive behavioural measure of IU in the general population [see also 3, 37]. It remains to be seen whether different adaptations of the task may be more sensitive, as IU was found to predict information gathering in a study comparing small samples of low and high compulsive individuals [26]. Inspection of results from this information gathering task suggested age may a moderating factor, at least in patients as no group differences were noted in an older sample [6, 13]. It is possible that over prolonged time, patients could increasingly forgo strategies to gather more information, thus reducing the sensitivity of such behavioural tasks. Present results however do not support this, as age did not correlate with information gathering indices.

Initial positive results in Condition 2 closely replicated previous findings, seemingly indicating less certainty in OCD patients [16]. However, we noted that evidence has emerged from studies employing the Beads task in schizophrenia revealing that some patients and to a lesser degree healthy volunteers miscomprehend instructions and do not perform the task as intended, particularly in Condition 2 [34]. Specifically, participants change their rating dramatically given a single bead of one colour after a long run in another. This marked over-adjustment appears tightly linked to miscomprehension [34]. When participants in the present samples who showed such evidence of miscomprehension were removed, the group differences between OCD patients and controls disappeared. This strongly suggests that previous positive findings in Condition 2 could have resulted from miscomprehension, particularly given our attempt to replicate and improve previous methodology. The evidence for miscomprehension in a subset of patients could be construed as a limitation of the current study, but does not detract from the main conclusion given that there were no substantial differences in information gathering performance in Condition 1 and in the sizeable remaining cohort in Condition 2. The finding does also emphasize the importance of assessing comprehension of the task. This does not appear to have been consistently considered in past studies on OCD. While some have stressed the importance of understanding the task [22], it is not clear how this was implemented. At least one study reported that some subjects dropped out because they found the task difficult to understand [16]. Careful task setup and stimulus presentation, training, testing for comprehension and inspection of the results should all be used routinely in future studies to address this concern.

OCD patients here, despite being largely free of any comorbidities, had significantly higher BIS attention-related impulsivity, consistent with previous findings [22, 23, 57]. Nevertheless, there was no evidence of a JTC reasoning style, or any association between impulsivity and task performance. Closer inspection of the items purported to capture attention impulsivity clearly indicates that individuals experiencing OC and anxiety symptoms would be expected to score high on many of them (e.g., ‘I often have extraneous thoughts when thinking’, ‘I have racing thoughts’, ‘I concentrate easily’) raising doubts about the discriminative validity of this subscale. In any case, ADHD patients, who are impulsive, do not show a JTC style or performance differences in the Beads task [58], further indicating that the task is not sensitive to decisional impulsivity. At the same time these results also clearly challenge the notion that those with high anxiety may shorten the duration of uncertainty [38], with no clear association between anxiety and performance noted.

The present study set out to replicate the most commonly used beads configuration, using only the 85:15 bead ratio. This may limit the generalizability of the results, though beads ratio did not previously seem to influence the task sensitivity to group differences [21, 37]. Similarly, the procedure was chosen to resemble most previous studies in an effort to bring clarity to the literature. While changing affective and motivational factors may increase its sensitivity, the initial appeal of the task was that it controlled for these factors, aiming to capture the difficulties OCD patients report in neutral contexts [17]. The study also did not include a comparison group of patients such as with anxiety disorders. However, the presence of such a group would not have altered the conclusion that the task is insensitive to high levels of OC symptoms and IU.

In sum, the results do not support the notion that patients with OCD seek additional information in the Beads task, nor that they adopt a JTC style. The results further indicate that the task should not be considered as a behavioural index of IU. Alternative approaches may be better pursued, such as with tasks assessing information gathering or certainty seeking behaviours where preliminary evidence supports some associations with OC and IU self-report measures [13, 54, 59]. Information gathering tasks typically consist of multiple trials and allow participants to gather as much information as they wish on each trial before making a decision or even allowing them to go back and gain additional information after making their decision [13, 54]. Certainty seeking tasks assess the extent participants will try to minimize ongoing states of uncertainty [59]. Such tasks can offer more fine-grained and potentially more sensitive measures whilst participants are under a more prolonged state of uncertainty.

## Supporting information

S1 File Supplementary information including S1 Text, S1A and S1B Tables. (DOCX)
