# Supplementary material for: Avoid jumping to conclusions under uncertainty in Obsessive Compulsive Disorder
Source: PLoS One. 2020 Jan 15;15(1):e0225970. doi: 10.1371/journal.pone.0225970 (PMC6961894; doi:10.1371/journal.pone.0225970)
Supplement: S1 File — (DOCX) [file pone.0225970.s001.docx]

**Supplementary Materials**

**Additional Analyses**

**Analyses of participants excluded from Condition 2**

We assessed whether those excluded from the primary analyses differed in demographic, clinical characteristics or in performance on Condition 1. While Study 1 HC did not show any differences, excluded patients were more likely to be female (11 females versus 4 males). Excluded patients also tended to be slightly slower in requesting beads, and more anxious but these differences did not survive correction for multiple comparisons. Excluded participants in the Study 2 sample had lower verbal IQ (*t*(123)=2.78, *p*<.001) and impulsivity (t(69)=2.12, p=.034). Though they did not differ in beads requested, they were slower to request them (*t*(123)=2.21, *p*=.029) and less accurate in choosing jar x (χ^2^(1)=13.26, p<.001; 99.07% vs. 82.35%, for retained versus excluded participants, respectively). The differences in Condition 1 performance remained significant after Bonferroni correction for 15 comparisons. S1 Table shows the patient and control sample characteristics for the sample retained in Condition 2.

**Analyses of Condition 2 of all participants**

For the Study 1 sample an ANOVA assessing mean probabilities estimated for each draw of condition 2 by group and jar indicated there was a significant interaction, such that draws departed more from the Bayesian norm for patients compared to controls and this varied as the draws progressed F(19,1862)=2.41, p<.001. Patients had lower estimates compared to controls for jar X, specifically from draw 11 onwards, F(19, 1862)=2.35, p<.001. Additionally, they had higher estimates compared to controls for the alternate jar, again this was more pronounced from draw 11. For the student sample of Study 2 a similar analysis indicated no significant effects for low- versus high-OCI participants *F*(19,2337)=.48, *p*=.97.

**S1 Table. Means and standard deviations of control and OCD patient group characteristics of patients included in Condition 2 analyses,**

|  |  | Controls  (n=40) | OCD  (n=34) |  |  |
| --- | --- | --- | --- | --- | --- |
| Characteristic | Measure | M (SD) | M (SD) | *Z* | *p* |
| Gender | M:F | 20:20 | 23:12 |  |  |
| Age | Years | 38.473 (14.83) | 40.14 (15.12) | 0.38 | 0.71 |
| Verbal IQ | NART | 115.74 (7.70) | 115.07 (6.37) | 0.34 | 0.74 |
| Obsessions & Compulsion | YBOCS |  | 19.80 (5.02) |  |  |
| Depression | MADRS |  | 6.17 (3.12) |  |  |
| Impulsivity- attention | BIS- attention | 14.55 (3.38) | 17.38 (4.66) | 3.10 | <0.002 |
| Impulsivity – motor | BIS - motor | 22.15 (3.68) | 20.29 (5.21) | 1.72 | 0.09 |
| Impulsivity – non planning | BIS – non planning | 24.25 (4.52) | 22.59 (6.08) | 1.77 | 0.08 |
| State Anxiety | STAI-S | 32.03 (9.86) | 41.34 (12.42) | 3.56 | <0.001 |
| Trait Anxiety | STAI-T | 36.95 (10.11) | 55.11 (11.54) | 5.65 | <0.001 |
| IU27 | IU | 55.60 (16.61) | 81.89 (22.68) | 4.89 | <0.001 |
| Prospective IU | IU | 17.35 (4.70) | 23.42 (6.61) | 4.02 | <0.001 |
| Inhibitory IU | IU | 8.25 (3.50) | 13.73 (4.63) | 4.85 | <0.001 |
| Obsessions & Compulsion | OCI | 10.25 (7.73) | 32.03 (11.60) | 6.45 | <0.001 |

Note. NART: National Adult Reading Test; YBOCS: Yale-Brown Obsessive Compulsive Scale; MADRS: Montgomery-Asberg Depression Rating Scale; BIS: Barret Impulsivity Scale; STAI-S: State/Trait Anxiety Inventory-State ; STAI-T: State/Trait Anxiety Inventory-Trait; IU: Intolerance of Uncertainty; OCI: Obsessive Compulsive Inventory-Revised; NART scores available for 34 controls and 29 patients. MADRS scores available for 29 patients.

**S2 Table. Dependent variables and *p* values for Condition 2 for all participants.**

|  | Study 1 | |  | Study 2 | |  |
| --- | --- | --- | --- | --- | --- | --- |
|  | Controls (n=50) | OCD (n=50) | *p* | Low OCI  (n=62) | High OCI  (n=63) | *p* |
| Initial posterior estimate (4) | 68.96 (17.39) | 75.20 (16.66) | .07 | 75.52 (13.83) | 73.08 (16.33) | .37 |
| Number of draws to certainty (5)** | 6.6 (4.85) | 5.58 (3.31) | .15 | 5.47 (3.10) | 5.86 (3.97) | .55 |
| Confirmatory effect (6a) | 7.56 (10.98) | 3.24 (15.43) | .11 | 8.65 (10.32) | 6.54 (12.49) | .31 |
| Disconfirmatory effect (6b) | 8.96 (22.85) | 17.92 (36.95) | .15 | 8.68 (21.68) | 4.51 (21.83) | .29 |
| Errors at draw ten (7)* | 22.00% | 20.00% | .81 | 6.45% | 19.05% | .04 |
| Number of draws from ten to change (8) | 2.31 (2.21) | 1.60 (1.42) | .07 | 2.20 (1.53) | 2.47 (1.83) | .23 |
| Size of first estimate change (9) | 18.22 (25.29) | 24.89 (31.66) | .11 | 7.33 (21.05) | 13.16 (25.83) | .19 |
| Final decision (10)* | 76.00% | 80.00% | .63 | 83.87% | 80.95 | .67 |
| Mean time in seconds per draw decision (11) | 12.78 (4.24) | 15.27 (8.97) | .07 | 11.80 (5.34) | 10.81 (3.49) | .22 |

Note. *Percentage of participants, **Sample size for this measure were 44 and 43 for controls and patients, respectively; 60 and 56 for low- and high-OCI participants, respectively.
